# Chiropractors’ attitudes toward drug prescription rights: a narrative review

**DOI:** 10.1186/s12998-014-0034-7

**Published:** 2014-09-24

**Authors:** Peter Charles Emary, Kent Jason Stuber

**Affiliations:** Private Practice, 201C Preston Parkway, Cambridge, ON N3H 5E8 Canada; Division of Graduate Education and Research, Canadian Memorial Chiropractic College, 6100 Leslie Street, Toronto, ON M2H 3 J1 Canada

**Keywords:** Knowledge, Attitudes, Drug prescriptions, Chiropractic

## Abstract

**Background:**

The right to prescribe drugs remains a contentious issue within the chiropractic profession. Nevertheless, drug prescription by manual therapy providers is currently an important topic. Notably, physiotherapists in the United Kingdom were recently granted limited independent prescribing rights. Reports suggest that physiotherapists in Australia now want those same rights, and as such a review of chiropractors’ general attitudes toward drug prescription is needed.

**Objective:**

To examine the literature concerning chiropractors’ attitudes toward drug prescription rights and to compare the opinions of chiropractors currently licensed to prescribe medication with those in the profession who are not.

**Methods:**

This was a narrative review, consisting of a formal literature search and summary of included articles. Electronic databases searched included the Cumulative Index to Nursing and Allied Health Literature, PubMed, and the Index to Chiropractic Literature. Inclusion criteria consisted of prospective studies published in English in peer-reviewed journals. Studies were required to contain data on chiropractors’ opinions toward medication prescription rights.

**Results:**

Of 33 articles identified, a total of seven surveys were included in the review. Of these, there was a general split in opinion among chiropractors regarding the right to prescribe drugs in chiropractic practice. Those supportive of prescribing rights favoured a limited number of over-the-counter and/or prescription-based medications such as analgesics, anti-inflammatories, and muscle relaxants. When questioned on full prescribing rights, however, chiropractors were generally opposed. In jurisdictions where chiropractors are currently licensed to prescribe from a limited formulary, such as in Switzerland, the majority perceived this right as an advantage for the profession. Moreover, continuing education in pharmacology was viewed as a necessary component of this privilege.

**Conclusions:**

Based on the literature to date there is a general split in chiropractors’ attitudes toward drug prescription rights. This split is most pronounced in countries where chiropractors are not licensed to prescribe medications. Notwithstanding, this is an important topic in chiropractic currently and warrants both further discussion and research to determine future directions and the implications of either pursuit or denial of prescription rights by chiropractors. Future surveys and/or qualitative studies of other chiropractors’ opinions toward gaining prescription privileges would be timely.

## Introduction

Throughout most of its nearly 120-year history the chiropractic profession has presented itself as a drugless, non-surgical healing profession. Since 1999, the World Federation of Chiropractic (WFC) has maintained the following policy statement on the use of prescription drugs: *“…for reasons of chiropractic principle, patient welfare and interdisciplinary cooperation the practice of chiropractic does not include the use of prescription drugs, and chiropractic patients who may benefit from prescription drugs should be referred, where appropriate, to a medical doctor or other suitably qualified health care practitioner”* [1]. From the WFC ‘Consultation on Identity’ survey in 2004, positioning of the profession as ‘non-drug, non-surgical health care’ was viewed by chiropractors worldwide as integral to how the profession should be perceived by the general public [2]. Regardless, contention over the right to prescribe drugs in chiropractic has existed in some countries for decades [3–14].

In or about 1989, four chiropractic-licensing boards in the United States (including Illinois, Florida, Vermont, and Texas) were actively lobbying for drug prescription rights [3]. In response to concerned members who had defected to the International Chiropractors Association (ICA), the American Chiropractic Association (ACA) set out to clarify its position on pharmaceuticals in chiropractic practice [4]. At their June 1989 national convention, the ACA delegates voted “decisively” to insert the following passage into their ‘Master Plan’: *“Chiropractic is a drug-free, non-surgical science and, as much, does not include pharmaceuticals or incisive surgery”* [4]. However, the ACA would not promote legislation to prohibit chiropractic state laws from including drug prescription within their respective scopes of practice [3,4]. As a consequence, the profession was further polarized on this issue in the United States [3–9]. Numerous commentaries on this topic have appeared in the peer-reviewed literature, both in favour of [10–14] and against [3–10] the prescription of drugs by chiropractors.

Currently, there are only two jurisdictions in the world where drug prescription has been incorporated into the scope of chiropractic practice. Since 1995 chiropractors in Switzerland have been licensed to prescribe from a limited formulary of over-the-counter (OTC) non-steroidal anti-inflammatory drugs (NSAIDs) and analgesics [15,16], while chiropractors in New Mexico, USA gained similar privileges in 2009 [17] (Table 1). Recently, other health care professions have also been gaining prescriptive rights. In a landmark ruling on July 24, 2012 (after nearly a decade of campaigning), physiotherapists in the United Kingdom were granted limited independent prescribing rights [18]. They joined the nursing, pharmacy, optometry, and podiatry professions who have been given similar privileges. It appears that Australian physiotherapists may also wish to expand their scopes of practice to include drug prescription rights as well [19].

**Table 1:** **Chiropractic formularies in Switzerland** [16] **and New Mexico, USA** [17]

**Switzerland**	**New Mexico, USA**
Analgesics	Hormones for topical, sublingual, oral use
• acetaminophen	• estradiol
• becetamol	• progesterone
• ben-u-ron	• testosterone
• dafalgan	• desiccated thyroid
• dolprone 500	Muscle relaxers; cyclobenzaprine
• minalgin	NSAIDs – prescription strength
• Novalgin	• ibuprofen
• treuphadol	• naproxen
NSAIDs	Prescription medications for topical use
• aspirin	• NMDC Ca^2^ dextromethorphan
• ibuprofen	• NSAIDs (ketoprofen, piroxicam, naproxen, ibuprofen, diclofenac)
• naproxen	• muscle relaxants; cyclobenzaprine
• diclofenac	• sodium channel antagonist; lidocaine
• piroxicam	
• indomethacin	Homeopathics requiring prescription
	Other substances by injection
	• sterile water
	• sterile saline
	• sarapin or its generic
	• caffeine
	• procaine HCL
	• epinephrine
	• homeopathic for injection
	Glutathione for inhalation

The purpose of this review is to examine the literature regarding chiropractors’ attitudes toward the inclusion of drug prescription rights in the chiropractic scope of practice. This review also seeks to compare the opinions of chiropractors currently licensed to prescribe medication with those in the profession who are not. The results of this study are important as they may have implications regarding future research and policy discussion relating to this topic. For the purposes of this review, *drugs* or *medicines* are defined as prescription and/or OTC pharmaceuticals, and do not include vitamins, minerals, or other nutritional supplements or homeopathic remedies.

## Review

### Methods

#### Search strategy

A search was conducted of the Cumulative Index to Nursing and Allied Health Literature (CINAHL), PubMed, and the Index to Chiropractic Literature (ICL) databases, without time limits to October 19, 2013. The searches were limited to human studies published in English in peer-reviewed journals. In all three databases, the truncated word ‘chiropract*’ was combined using the Boolean operator, ‘*AND*’ with a variety of medical subject headings (MeSH) and text words relevant to the topic (see Appendix 1 for the complete search strategy). ‘*Switzerland*’ and ‘*New Mexico*’, the two jurisdictions where chiropractors are currently licensed to prescribe medication, were also used as key words in the database searches. Reference searching of any retrieved articles was undertaken, as was personal contact with authors known to have expertise on this subject to determine if they were aware of potentially relevant references.

#### Article selection criteria

To select potentially relevant papers, the retrieved citation titles were scanned, followed by a review of their abstracts. Full manuscripts of those that appeared to meet the review criteria were then retrieved for further analysis. The inclusion and exclusion criteria for this review are displayed in Table 2.

**Table 2:** **Inclusion and exclusion criteria**

**Inclusion criteria**	**Exclusion criteria**
• Articles of any type of prospective research design,	• Articles published in trade magazines,
• Articles published in English,	• Letters to the Editor, Commentaries,
• Articles published in a peer-reviewed journal, and	• Articles not published in English, and/or
• Articles that contain data on chiropractors’ opinions toward medication prescription rights.	• Articles not specific to the topic of chiropractors’ opinions toward medication prescription rights.

### Appraisal methods

As this was a narrative review, included articles were not evaluated for their quality. However, data was extracted and presented in tabular form. The categories depicted included study location, survey method, number of respondents and response rate, percentage in favour of prescription rights, and percentage opposed to prescription rights. Data pertaining to chiropractic prescribing rights in Switzerland was presented in a separate table, with the last category consisting of attitudes toward existing prescription rights and its application in practice.

### Synthesis of the results

As this was a narrative review, the results were not to be synthesized.

## Results

### Search results

Thirty-three potential articles were identified through the literature searches and retrieved for full paper review. Twenty-two of these articles were identified from literature searches, eight from reference searching, two from secondary searching of unpublished literature, and one from personal contact with authors known to have expertise in this area.

### Included articles

A total of seven articles, all surveys, were deemed includable in the review [15,20–25]. Reasons for exclusion of the 26 excluded papers are presented in Table 3, and a summary of the included non-Swiss and Swiss articles is presented in Tables 4 and 5, respectively.

**Table 3:** **Reasons for excluding papers from this review**

**Reason**	**Reference**
Commentary from a trade magazine	[26–29]
Not about chiropractors’ attitudes toward prescribing rights in chiropractic	[11,30–36]
Commentary from a peer-reviewed publication	[3–10]
Letter to the Editor	[12–14]
Not published in a peer-reviewed journal	[2,37,38]

**Table 4:** **Summary of non-Swiss studies reviewed**

**First author, year of publication**	**Location**	**Survey method**	**Number of respondents and response rate (%)**	**Percentage in favour of prescription rights**	**Percentange opposed to prescription rights**
Jamison [20], 1991	Australia	Postal	339 respondents, 20% response rate	• 2% of respondents felt that prescription drugs should “frequently” be used in chiropractic practice, 31% indicated “sometimes,” and 25% indicated these should be used “rarely”	• 42% of respondents felt that prescription drugs should “never” be used in chiropractic practice
				• Of those supportive of drugs, 84% favoured NSAIDs, 80% favoured analgesics, and 74% favoured muscle relaxants	• 13% indicated that they “never” advise acute patients to take analgesics
				• 59% of all respondents indicated that they advise acute patients to take analgesics “always,” “usually,” or “sometimes,” while an additional 28% do so at least “rarely”	• 27% indicated that they “never” suggest acute patients take NSAIDs (while the remaining 7% of respondents queried the meaning of the abbreviation ‘NSAIDs’)
				• 42% of all respondents indicated that they suggest acute patients take NSAIDs “always,” “usually,” or “sometimes,” while an additional 24% do so at least “rarely”	• Of those supportive of drugs, 65% were opposed to chiropractors prescribing antibiotics, 71% to steroids, and 74% to anti-hypertensive prescription by chiropractors
Jacobson [21], 1999	Oklahoma, USA	Postal	304 respondents, 49% response rate	• 54% favoured prescription rights for chiropractors	• 28% of respondents “never” recommended OTC drugs to patients in their chiropractic practice
				• 13% of respondents “often” recommended OTC drugs to patients in their chiropractic practice, 26% did so “sometimes,” and 33% recommended OTC drugs at least “seldomly”	
Wilson [22], 2003	United Kingdom	Postal	816 respondents, 58% response rate	• 36% of respondents felt that chiropractors should be allowed to prescribe medications on a restricted basis (e.g. mild analgesics, NSAIDs, and muscle relaxants)	• Data not reported
McDonald [23], 2004	North America wide – random sample	Postal	687 respondents, 63% response rate	• 54% favoured chiropractors writing OTC drug prescriptions	• 51% were opposed to chiropractors writing prescriptions for musculoskeletal medicines (e.g. muscle relaxants)
					• 89% were opposed to chiropractors writing prescriptions for any and all medicines, including controlled substances
Pollentier [24], 2007	United Kingdom – random sample	Postal	263 respondents, 54% response rate	• 28% of respondents felt it would be beneficial if chiropractors were allowed to prescribe medication on a restricted basis (e.g. mild analgesics, NSAIDs, and muscle relaxants)	• 59% felt that limited prescription rights would not be beneficial

**Table 5:** **Summary of Swiss studies reviewed**

**First author, year of publication**	**Location**	**Survey method**	**Number of respondents and response rate (%)**	**Attitudes toward existing drug prescription rights and its application in practice**
Robert [15], 2003	Switzerland	Postal	126 respondents, 51% response rate	• 61% of respondents prescribe OTC medications (i.e. analgesics and NSAIDs) to their patients
				• 82% consider these limited prescribing rights a privilege for the chiropractic profession in Switzerland
				• 76% would like this privilege extended to include some prescription-based analgesic and muscle relaxant medications
Wangler [25], 2010	Switzerland, Berne canton	E-mail	36 respondents, 77% response rate	• 72% of respondents agreed that the ability to prescribe OTC analgesic and anti-inflammatory medications was an advantage for the chiropractic profession in Switzerland
				• 58% would like this privilege extended to include some additional prescription-based NSAIDs, muscle relaxants, and analgesics
				• 41% perceived medication as a necessary component of treatment (e.g. to help patients who cannot sleep because of pain and to speed up recovery)
				• 91% agreed that continuing education in pharmacology was necessary

### Main outcomes

Based on the literature to date there is a general split in opinion among chiropractors regarding the right to prescribe drugs in chiropractic practice. This split in opinion is most pronounced in countries where chiropractors are not currently licensed to prescribe medications (Table 4). For instance, in a 1990 survey involving members of the Federal Branch of the Australian Chiropractors’ Association [20], 42% of respondents felt that prescription drugs should “never” be used in chiropractic practice. This was compared to 58% who believed that prescription drugs should “frequently” (2%), “sometimes” (31%), or at least “rarely” (25%) be used. In 1997, Jacobson and Gemmell [21] conducted a survey of all registered chiropractors in Oklahoma, USA. Out of 304 total respondents, 54% felt that chiropractors should have the right to prescribe prescription drugs. Similarly, McDonald *et al.* [23] surveyed chiropractors from various North American regions and found that 54.3% of respondents felt they should be permitted to write OTC prescriptions, while a nearly equal number (51.2%) were opposed to prescription-based musculoskeletal medicines (e.g. muscle relaxants). In the United Kingdom, Wilson [22] and Pollentier and Langworthy [24] found that 36% and 28% of respondents, respectively, were in support of chiropractors prescribing medications on a restricted basis (e.g. mild analgesics, NSAIDs, and muscle relaxants). However, Pollentier and Langworthy found that 59% of their respondents did not consider this proposition to be beneficial [24]. Therefore based on those surveyed, chiropractors in Australia, North America, and the United Kingdom appear to be divided in their attitudes toward drug prescription rights.

Conversely, the general attitude of chiropractors who are already *licensed* to prescribe medications is much different (Table 5). Since 1995, chiropractors in Switzerland have been able to prescribe from a limited formulary of OTC drugs and topical medications (see Table 1). In 1999, Robert [15] undertook a survey of all Swiss chiropractors to assess their general attitude towards this limited privilege, as well as the possibility of extending it to include some prescription-based medicines. Of 126 respondents, 61% indicated that they prescribed OTC medications (i.e. analgesics and NSAIDs) to their patients; and 82% considered these limited privileges an advantage for the chiropractic profession in Switzerland. Moreover, 76% wanted these privileges extended to include some prescription-based analgesic and muscle relaxant medications. In 2010, Wangler *et al.* [25] conducted a survey of all chiropractors in Berne, Switzerland. Similar to the Robert [15] survey, the majority of respondents (72%) agreed that the ability to prescribe OTC analgesic and anti-inflammatory medications was an advantage for chiropractors in Switzerland, while 58% also wanted this privilege extended to include some additional prescription-based NSAIDs, muscle relaxants, and analgesics. Fewer respondents (41%) perceived medication as a necessary component of chiropractic treatment, mainly using them to help patients who cannot sleep because of pain and/or to help speed up recovery. Finally, a large majority (91%) agreed that continuing education in pharmacology was a necessary component to the privilege of drug prescription in chiropractic. Overall the general attitude of chiropractors surveyed in Switzerland appears to be more favourable towards drug prescription when compared to chiropractors surveyed in other regions.

The results of three of the surveys indicate that among chiropractors who are supportive of prescribing rights, a limited number of OTC and/or prescription medications are favoured (see Table 4). For example, of Australian chiropractors who were supportive of drug prescription, 84% favoured NSAIDs, 80% analgesics, and 74% muscle relaxants [20]. This view was also supported by chiropractors in Oklahoma [21] where more than half (56.7%) of those in favour of prescribing rights indicated that only a select group of drugs such as analgesics, anti-inflammatories, and muscle relaxants, should be permitted for prescription by chiropractors. In North America [23], an overwhelming majority (88.6%) of respondents, whether supportive of drug prescription by chiropractors or not, were opposed to *full* prescribing rights (i.e. the ability of chiropractors to write prescriptions for any and all medicines, including controlled substances). As such, whether practising in Switzerland or elsewhere, chiropractors with attitudes favourable toward drug prescription surveyed in these regions tend to prefer limited prescribing rights within a musculoskeletal scope of practice.

Two of the surveys from this review detail the use of OTC medications in chiropractic practice (see Table 4). In Oklahoma, USA [21], for example, 28% of survey respondents indicated that they “never” recommended non-prescription drugs to their patients. However, 72% admitted that they did so “often” (12.9%), “sometimes” (26.5%) or at least “seldomly” (32.6%). Australian chiropractors’ behaviours were similar when it came to the use of OTC medicines [20]. Thirteen percent of respondents “never” advised acute patients to take analgesics, while 59% did so “always,” “usually,” or “sometimes,” and an additional 28% advised these drugs at least “rarely.” Regarding NSAIDs, 27% “never” suggested these to acute patients, while 42% “always,” “usually,” or “sometimes” did, and an additional 24% suggested NSAIDs at least “rarely.” Thus, although chiropractors in these regions appear to be divided in their attitudes toward prescribing rights in general, many often still recommend OTC medications to patients in practice.

## Discussion

The main finding of this narrative review was that based on the literature to date there is a general split in chiropractors’ opinions regarding drug prescription rights in chiropractic. For instance, from published surveys [20–24] of chiropractors in Australia, the United States, Canada, Mexico, and the United Kingdom, no more than 58% of respondents were in favour of chiropractic prescribing rights and no less than 42% were opposed. In some of these studies, this split was nearly even [21,23]. In Switzerland on the other hand, the majority (72% to 82%) perceived their prescriptive privileges as an advantage for the profession [15,25], and between 58% and 76% wanted to see these privileges extended to include some additional prescription-based analgesic and muscle relaxant medications. These divided attitudes within the chiropractic profession stand in stark contrast to the views of those in other allied health care professions who are unified on this issue [39–41]. To better understand this chiropractic split, therefore, the implications of drug prescription for the profession must first be considered.

There are some concerns that are often mentioned by authors who are against chiropractors gaining access to prescription rights. One of the most pressing concerns is the lack of chiropractic education and training in pharmacology and toxicology [7,8,10]. In addition, there is concern about the risk of losing chiropractic’s distinct professional identity [4–9]. The first concern is legitimate, in that the basic chiropractic educational curriculum contains only 12 hours of coursework in pharmacology [42], although the actual number of course hours may vary from educational institution to institution. Nevertheless, further undergraduate and/or post-graduate education and training would be required for those in the profession wishing to prescribe medications. As for chiropractic’s identity, many have argued that the profession has always *lacked* a clear identity, resulting in its failure to establish full cultural authority and respect within mainstream society [43–45]. Some of these same authors [43] have also warned of the U.S. osteopathic experience, where given the option of prescribing drugs and using other more invasive interventions, the role of manipulative therapy has diminished (and nearly vanished) from this profession.

Another issue to consider is that of interprofessional relations. Some have argued that the inclusion of prescription drugs within the chiropractic scope of practice would negatively impact relationships with the medical profession [46]. However, if working as part of a collaborative team this expanded role for chiropractors may be supported by physicians, particularly if limited to treating musculoskeletal conditions [30,47]. In Switzerland, chiropractors are among one of five government-recognized medical professions (i.e. human medicine, chiropractic medicine, veterinary medicine, dentistry, and pharmacology) [16,30]. Swiss chiropractors have limited prescribing rights and are integrated and accepted by the medical community [30]. In the United Kingdom, the recent provision of limited prescribing rights to physiotherapists and podiatrists has also been met with support from medical doctors [18].

One important published commentary on this topic was written in 1999 by Australian chiropractor, Dr. Jennifer Jamison [10]. In the article, Dr. Jamison highlighted both the potential benefits and detriments of drug prescription in chiropractic. On one hand, limited prescribing rights would give chiropractors and their patients more direct access to prescription analgesic and anti-inflammatory medications, allowing for a more complete management approach to patients with musculoskeletal conditions, a statement supported by some evidence [48,49]. Unless restricted to certain medications, these privileges could also give chiropractors access to antibiotics and other drugs used in treating chronic complaints (e.g. hypertension, anxiety, and depression). Conversely, these same privileges could fundamentally change the focus of chiropractic care from health to disease, increase the burden of practitioner responsibility in terms of recognizing and monitoring drug side effects, and create the need for additional chiropractic undergraduate training and practitioner self-education [10]. Further implications would include necessary regulatory and legislative changes, consideration of legal and ethical issues, and increases to chiropractic malpractice/liability insurance coverage [47].

The aforementioned split in opinion over chiropractic prescribing rights could be reflective of differences in philosophical orientation. For example, McDonald *et al*. [23] found that out of 647 respondents, 34.3% of North American chiropractors surveyed classified themselves as practising within a “broad” scope of practice (i.e. the often described chiropractic ‘mixer’), 19.3% were “focused scope” (or the often described ‘straight’ chiropractors), and the remaining 46.4% identified themselves somewhere inbetween. Among the broad scope respondents, more than three quarters (77.1%) believed that chiropractors should be permitted to prescribe OTC medications, while only 17.6% of the focused group felt the same way. These philosophical divisions within the chiropractic profession have also been shown by others [50].

The split in opinion over prescribing rights could also possibly be explained by the fact that, at least in the McDonald *et al*. survey, North American chiropractors as a group were of the opinion that only 39.8% of all pharmaceutical prescriptions filled annually were clinically beneficial [23]. This type of attitude might again be more reflective of philosophical orientation, versus that of evidence-based practice. Regardless, as suggested by McDonald *et al*. [23], the legal right to prescribe certain drugs to patients in chiropractic practice would include the right of *un*-prescribing these same drugs. For example if given limited prescriptive authority, this would enable chiropractors to advise patients against overuse and over-reliance on medications such as analgesics, NSAIDs, and muscle relaxants [25,46]. Therefore, chiropractors may not all agree on prescribing rights in chiropractic, but perhaps all could unite on the issue of prescribing rights related to counselling patients on medication use?

Another finding of this review was that despite the profession’s split over the right to prescribe drugs, many chiropractors often still recommend OTC medications to their patients. For instance, while only 54% of respondent chiropractors in Oklahoma, USA [21] were supportive of prescribing rights, 72% indicated that they recommended non-prescription drugs with variable frequency in clinical practice. Similarly, Australian chiropractors [20] were split over drug use in chiropractic, however 87% and 66% of respondents still advised acute patients to take OTC analgesic and NSAID medications to some extent, respectively. Interestingly, chiropractors in Switzerland are licensed to prescribe OTC medications in practice, yet they appear to do so with less frequency than their international colleagues. For example, when asked how often Swiss chiropractors prescribed analgesics, NSAIDs, and muscle relaxants [25], respondents indicated that only 60%, 55.9%, and 21.5% did so, respectively. Similarly, as part of a 2009 ‘Job Analysis’ survey Humphreys *et al*. [30] asked Swiss chiropractors how often they prescribed pain medication in practice. The majority (70%) indicated that they did so for between 0% and 25% of patients, with an additional 15% prescribing these for 26% to 50% of their patients. Only 2% of Swiss chiropractors prescribed pain medication for 51% to 75% of patients, and none (0%) did so for between 76% and 100% of their patients. Therefore, although Swiss chiropractors are generally more positive in their attitudes toward drug prescription, the majority utilize this privilege on a selective basis with their patients. This reduced usage could possibly be explained by differences in training and continuing education, particularly in pharmacology, that Swiss chiropractors receive [25,30] when compared to others in the profession [42].

Aside from Switzerland, the second jurisdiction worldwide where chiropractors can currently gain licensure to prescribe medications is in New Mexico, USA. Since March 31, 2009, chiropractors certified as ‘advanced practice chiropractic physicians’ have been eligible to register for limited prescriptive authority through the New Mexico Board of Chiropractic Examiners [17]. The National University of Health Sciences (in Chicago, Illinois) currently offers a two-year Master of Science degree in ‘Advanced Clinical Practice’ which, among other courses, offers further training in pharmacology [51], allowing chiropractors to apply for ‘advanced practice’ status in the state of New Mexico, along with the use of its limited formulary (see Table 1). Those chiropractors in New Mexico wishing to renew their advanced practice certification must do so annually, completing an additional 10 hours of continuing education over-and-above their required hours for general chiropractic licensure in pharmacology, toxicology, and/or medication administration relevant to the current formulary [17]. Regarding drug prescription utilization, 146 (or approximately 30%) of the 480 licensed chiropractors currently practising in New Mexico are certified as advanced practice chiropractic physicians [52].

In Switzerland, there is also general support for expanding the current chiropractic formulary to include additional prescription-based analgesics (e.g. opioids) and muscle relaxants. In fact, the Swiss Chiropractic Association has recently put forth a proposal to the Swiss government, requesting this expansion; and it has been successful [53]. In New Mexico, chiropractors have similarly made attempts at expanding their current formulary (to include additional prescription drugs, as well as drugs to be administered by injection), but without success in large part due to opposition from the ICA [54]. This push for expanded prescribing rights in New Mexico appears to be motivated by a desire to train advanced practice chiropractors to operate as ‘primary care physicians’, particularly in rural areas where there is a current shortage of general medical physicians [36].

Concerning the issue of full prescribing rights, however, evidence from the reviewed literature suggests that chiropractors are generally opposed [20,21,23]. In the Jamison survey [20], for example, of the Australian chiropractors who were supportive of drugs (i.e. NSAIDs, analgesics, and muscle relaxants) there was almost equal opposition to the use of medications for non-musculoskeletal conditions; indeed, 65% were opposed to chiropractors prescribing antibiotics, 71% were opposed to steroid prescription, and 74% were opposed to anti-hypertensive prescription by chiropractors. Similarly in North America [23], despite a near even split on attitudes toward limited prescribing rights, a large majority (88.6%) of respondents were opposed to chiropractors having full prescribing rights (i.e. the ability to write prescriptions for any and all medicines). As such, previous attempts at expanding the New Mexico chiropractic scope of practice to beyond a limited number of medications [36,54], appears to be contrary to the general attitude of many others in the profession.

### Limitations

This review has several limitations. First, the literature search was limited to three databases, included only articles written in English, and excluded non-peer reviewed and/or non-scholarly publications such as those from trade magazines. Therefore, other pertinent articles or commentaries on the topic of drug prescription in chiropractic may have been omitted. Second, all of the seven papers included in this review were surveys. However, this study sought information about chiropractors’ general attitudes and opinions regarding drug prescription in chiropractic, and as such inclusion of this study design was appropriate. Third, several of the surveys reviewed were limited by low response rates [15,20–24]. Moreover, none of the authors from these surveys compared respondents with non-respondents. As such, if there were systematic differences as opposed to chance differences between respondent and non-respondent chiropractors surveyed in a given region, the results obtained would not be generalizable to the entire population of chiropractors in that region [55]. Therefore outside of Berne, Switzerland, the general attitude of chiropractors toward drug prescription in chiropractic remains somewhat in question. Because of this, further surveys and/or qualitative research studies are warranted in order to clarify the general attitude of chiropractors toward drug prescription in chiropractic both locally and internationally. In future surveys, non-response bias should be investigated, particularly if response rates obtained from these surveyed populations are low. Finally, when compared to other research designs (e.g. systematic reviews), narrative reviews of the literature are more susceptible to bias [56].

## Conclusions

This review identified seven surveys pertinent to the topic of chiropractors’ attitudes toward drug prescription in chiropractic published in the peer-reviewed literature. Of these, there was a general split in opinion concerning the right to prescribe medications. In jurisdictions where chiropractors are currently licensed to prescribe a limited number of medications, such as in Switzerland, the majority perceived this right as an advantage for the profession. Moreover, continuing education in pharmacology was viewed as a necessary component of this privilege. Future surveys and/or qualitative studies of chiropractors’ attitudes toward gaining similar privileges would be timely.

## Appendix 1. Literature search terms

### Search terms

- chiropract* + drug prescription
- chiropract* + medication prescription
- chiropract* + prescribing rights
- chiropract* + limited prescribing rights
- chiropract* + prescription writing
- chiropract* + pharmaceuticals
- chiropract* + prescriptions
- chiropract* + drugs
- chiropract* + non-prescription drugs
- chiropract* + over-the-counter medications
- chiropract* + Switzerland
- chiropract* + New Mexico

## Abbreviations

ACA: American chiropractic association
CINAHL: Cumulative index to nursing and allied health literature
ICA: International chiropractors association
ICL: Index to chiropractic literature
MeSH: Medical subject heading
NSAIDs: Non-steroidal anti-inflammatory drugs
OTC: Over the counter
US: United States
USA: United States of America
WFC: World federation of chiropractic
